# Supplementary material for: Integrated Multitrophic Aquaculture; Analysing Contributions of Different Biological Compartments to Nutrient Removal in a Duckweed-Based Water Remediation System
Source: Plants (Basel). 2022 Nov 15;11(22):3103. doi: 10.3390/plants11223103 (PMC9698553; doi:10.3390/plants11223103)
Supplement: Supplementary file 1 [file plants-11-03103-s001.zip › plants-2009974-supplementary.pdf]

**Before starting the experiment**			
	1	Placement of 6 tanks inside the canal for 7days to allow establishment of natural biofilm in the internal surface	
	2	Removal of tanks from the canal	
	3	Sun-drying of three tanks and scraping of dried biofilm from the internal surface of the tanks	
	4	Ashing of half of the biofilm scraped to determine the organic fraction	
	5	Analyses of the other half to quantify TN and TP in the initial biofilm	
	6	Burial of three tanks next the canal, leaving approximately 5 cm above the ground	
	7	Filling of each tank with 70 l of water pumped from the canal	
	8	Placement of approximately 800 cm ^{2} of duckweed mat, collected from the canal, in each tank	
		Collection, weighting, and drying of three samples of duckweed of equal area for the determination of indicative TN and TP in the initial biomass	
**Daily operations**			
8:00 am	1	Measurement of chlorophyll and cyanobacteria concentration	Repeated for 8 days
	2	Measurement of pH and water temperature	
	3	Collection of a water sample per tank	
	4	Removal of water from the tank using a pump, slowly, without disrupting the duckweed mat	
	5	Collection of 1 water samples from the canal	
	6	Refillmnent of tanks with 70 l of water pumped in from the canal	
8:00 pm	1	Measurement of Chlorophyll and cyanobacteria concentration	
	2	Measurement of pH and water temperature	
	3	Collection of 1 water sample per tank	
	4	Removal of water from the tank using a pump, slowly, without disrupting the duckweed mat	
	5	Collection of 1 sample of water pumped in from the canal	
	6	Refilling of tanks with 70 l of water pumped in from the canal	
**End of the experiment**			
	1	Collection of duckweed mat from the three tanks and drying for the determination of TN and TP	
	2	Removal of water from the tanks	
	3	Sun-drying of the tanks	
	4	Ashing of half of the biofilm scraped to determine the organic fraction	
	5	Analyses of the other half to quantify TN and TP in the final biofilm	

Table S1. Summary of the experimental protocol
